# Identification of a Thyroid Hormone Derivative as a Pleiotropic Agent for the Treatment of Alzheimer’s Disease

**DOI:** 10.3390/ph14121330

**Published:** 2021-12-19

**Authors:** Massimiliano Runfola, Michele Perni, Xiaoting Yang, Maria Marchese, Andrea Bacci, Serena Mero, Filippo M. Santorelli, Beatrice Polini, Grazia Chiellini, Daniela Giuliani, Antonietta Vilella, Martina Bodria, Eleonora Daini, Eleonora Vandini, Simon Rudge, Sheraz Gul, Michale O. J. Wakelam, Michele Vendruscolo, Simona Rapposelli

**Affiliations:** 1 Department of Pharmacy, University of Pisa, Via Bonanno 6, 56126 Pisa, Italy; massimiliano.runfola@farm.unipi.it (M.R.); andrea.bacci@phd.unipi.it (A.B.); 2 Centre for Misfolding Diseases, Department of Chemistry, University of Cambridge, Cambridge CB2 1EW, UK; mp717@cam.ac.uk (M.P.); xy292@cam.ac.uk (X.Y.); 3 Molecular Medicine, Istituto di Ricovero e Cura a Carattere Scientifico (IRCCS) Fondazione Stella Maris, Via dei Giacinti 2, 56128 Calambrone, Italy; maria.marchese2086@gmail.com (M.M.); s.mero27@gmail.com (S.M.); filippo.santorelli@fsm.unipi.it (F.M.S.); 4 Department of Pathology, University of Pisa, Via Savi 10, 56126 Pisa, Italy; b.polini@studenti.unipi.it (B.P.); 6grazia1@gmail.com (G.C.); 5 Department of Biomedical, Metabolic and Neural Sciences, University of Modena and Reggio Emilia, Via G. Campi 287, 41125 Modena, Italy; daniela.giuliani@unimore.it (D.G.); antonietta.vilella@unimore.it (A.V.); martina.bodria@unimore.it (M.B.); eleonora.daini@unimore.it (E.D.); eleonora.vandini@gmail.com (E.V.); 6 Ibabraham Research Campus, The Babraham Institute, Cambridge CB22 3AT, UK; Simon.rudge@babraham.ac.uk (S.R.); michael.wakelam@babraham.ac.uk (M.O.J.W.); 7 Fraunhofer Institute for Translational Medicine and Pharmacology ITMP, Schnackenburgallee 114, 22525 Hamburg, Germany; Sheraz.Gul@itmp.fraunhofer.de; 8 Fraunhofer Cluster of Excellence Immune-Mediated Diseases CIMD, Hamburg Site, Schnackenburgallee 114, 22525 Hamburg, Germany; 9 CISUP, Center for Instrument Sharing, University of Pisa, 56126 Pisa, Italy

**Keywords:** Alzheimer’s disease, polypharmacology, *C. elegans*, autophagy, drug discovery, zebrafish, 5XFAD mice

## Abstract

The identification of effective pharmacological tools for Alzheimer’s disease (AD) represents one of the main challenges for therapeutic discovery. Due to the variety of pathological processes associated with AD, a promising route for pharmacological intervention involves the development of new chemical entities that can restore cellular homeostasis. To investigate this strategy, we designed and synthetized SG2, a compound related to the thyroid hormone thyroxine, that shares a pleiotropic activity with its endogenous parent compound, including autophagic flux promotion, neuroprotection, and metabolic reprogramming. We demonstrate herein that SG2 acts in a pleiotropic manner to induce recovery in a *C. elegans* model of AD based on the overexpression of Aβ42 and improves learning abilities in the 5XFAD mouse model of AD. Further, in vitro ADME-Tox profiling and toxicological studies in zebrafish confirmed the low toxicity of this compound, which represents a chemical starting point for AD drug development.

## 1. Introduction

Alzheimer’s disease (AD) affects over 45 million people worldwide, a figure predicted to double by 2030 [1]. This condition is characterized by the deposition in brain tissues of amyloid plaques formed by the amyloid β peptide (Aβ) and of neurofibrillary tangles formed by tau protein. The accumulation of Aβ and tau aggregates induces a cascade of events leading to neuronal death along with the insurgence of cognitive deficit and motor symptoms [1]. Growing evidence suggests that the pathogenesis of AD is characterized by a tangled network of impaired mechanisms, including protein homeostasis disruption, neuro-inflammation, and lipid metabolism changes [2,3,4,5,6,7]. In particular, it is well established that defective autophagy is implicated in AD pathogenesis [8] and intracellular Aβ monomers negatively affect autophagy [9], highlighting once again the existence of a vicious circle in which accumulation of Aβ, decrease of autophagy flux and increase of inflammatory mechanisms due to metabolic imbalance contribute to different aspects of aging and to the progression of detrimental age-related diseases, such as AD. This pathological complexity represents a major barrier in the quest for AD therapies [10]. Despite the numerous attempts at identifying pharmacological treatments able to halt or slow down AD pathological conditions, drug discovery programs of the last three decades have produced limited results in terms of efficacy, which has been a poor payoff when considering the large amount of economic investment in this area [11]. The recent approval of aducanumab by the FDA has renewed the interest in the search for effective disease-modifying treatments [12]. One affordable approach could be the identification of polypharmacological small molecules capable of restoring healthy conditions by a multi-tiered action on different pathways [13]. To overcome the difficulties in identifying such pleiotropic drugs, re-integration of phenotypic screening into the current pipeline of drug development has been proposed [14]. Monitoring phenotypic responses of living organisms when exposed to small molecules could provide new disease-modifying pharmaceutical strategies against complex pathologies such AD. It is noteworthy that more than 1 out of 3 first-in-class drugs have been discovered through this approach between 1999 and 2008 [15].

In 2015, we reported the SG compounds as a new class of synthetic diphenylmethane thyronamine analogues [16]. Thyroid hormone (TH) signalling offers a large multi-tiered pharmacology acting on protein homeostasis, neuronal development, and metabolism reprogramming, among others. Moreover, the link between alterations of TH signalling and AD as well as other proteinopathies has been established and documented [17,18,19,20]. Further investigations allowed identification of SG2, as a drug-like small molecule with an interesting polypharmacological profile. We previously demonstrated that SG2 is capable of exerting pro-learning, anti-amnestic and pro-autophagic effects in rodents [21]. In particular, SG2 was found to produce a time-dependent recovery of autophagic activity in U87MG cells, due to the downregulation of mTOR [21]. Additionally, we showed the ability of SG2 to modulate lipid metabolism through the modulation of the AMPK/ACC pathway [22]. More recently, the protective effect of SG2 against neuronal plasticity impairment has been further confirmed in ex-vivo models of AD using mhAPP mice [23], suggesting that SG2 may have therapeutic potential in the treatment of AD.

In this study, we evaluated the potential of SG2 as a polypharmacological strategy for AD drug discovery by assessing: (i) its capability to extend the lifespan and promote autophagy in an in vivo model of AD of *C. elegans*, (ii) its safety on small vertebrate model, zebrafish (Danio rerio), and (iii) its protective effects during the prodromal phase of AD pathology in the 5XFAD mouse model of AD, by means of behavioural screening.

## 2. Results

### 2.1. SG2 Restores Motility in a C. elegans Model of AD

Given the positive results previously reported regarding SG2 activity against a plethora of physiological pathways involved in AD, we assessed whether the polypharmacological profile of SG2 could also exert beneficial effects in vivo for models of AD. To this end, we employed a *C. elegans* model of AD in which human Aβ42 is constitutively expressed in the big muscle cells, leading to an age- and temperature-dependent paralysis [24]. We used a treatment protocol that we had previously developed and validated against a large set of small molecules to delay the onset in AD models [25,26,27] in combination with a fully automated machine vision system that provide high-throughput worm tracking with high statistical significance [28,29].

**Figure 1:**
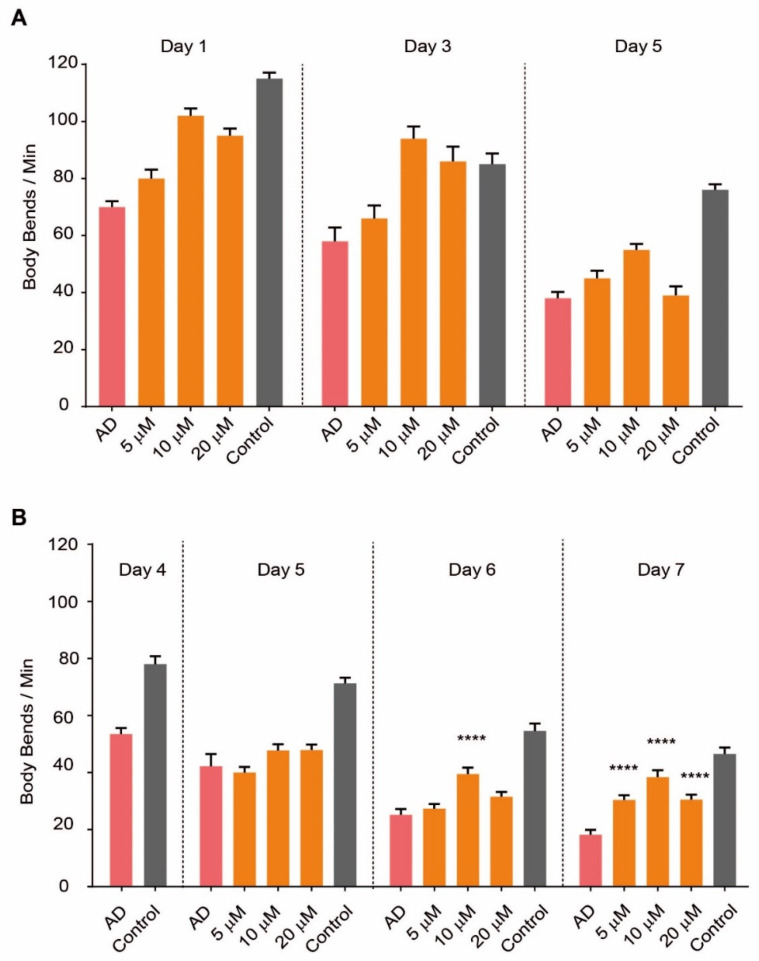
SG2 recovers fitness in a *C. elegans* model of AD. SG2 was administered to the worms following two different treatment regimes, namely early treatment at D0 (**A**) or late treatment at D4 (**B**). SG2 dosed at 5, 10 and 20 µM showed significant dose-dependent recovery effect, expressed as body bends/min, on AD worms (**A**,**B**). Error bars represent the standard error of the mean (SEM). For statistical tests, One-Way ANOVA was used. *p* ≤ 0.0001 (****).

We administered SG2 to *C. elegans* worms following two different treatment regimens, namely, early treatment at day zero of adulthood (D0) or late treatment at day 4 (D4), to mimic prevention and treatment, respectively (Figure 1A). By administering SG2 at 5, 10 or 20 µM we observed a significant dose-dependent recovery effect on AD worms when the compound was dosed at D0 (Figure 1A). Remarkably, we could also observe a recovery effect when the compound was dosed at later stages, when the plaques were already present, and the paralysis onset has occurred (Figure 1B); a maximum effect was recorded at day 7 (D7) for 10 and 20 µM. The effects became more significant after several days of treatment (Figure 1B), showing cellular recovery in the pathological model.

### 2.2. Studies on Aβ Aggregation Process In Vitro

**Figure 2:**
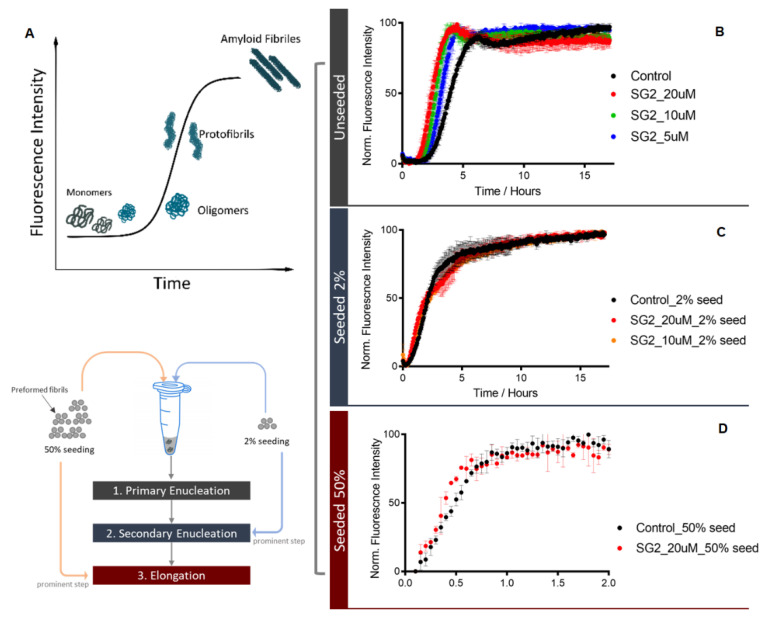
SG2 does not directly inhibit Aβ42 aggregation in in vitro assays. The aggregation of 2 µM Aβ42 was performed in 20 mM sodium phosphate buffer with 0.2 mM EDTA at pH 8. The amyloid-specific dye thioflavin T (ThT) at 20 µM was used as a fluorescence probe to monitor the progression of the aggregation process as depicted in panel (**A**). SG2 was added to Aβ42 at different concentrations in presence or in absence of preformed fibrils (seeds), as shown in each panel. In the seeded assays, 2% or 50% of preformed fibrils were added to the solution before the start of the aggregation process. Varying the seeds concentration in the assay solution allows to monitor different steps of the Aβ42 aggregation process. Results are presented as normalized fluorescence intensity, which is indicative of the Aβ42 formation, over time. SG2 has a slight effect on primary enucleation (unseeded assay, panel (**B**)), while does not affect the secondary enucleation (panel (**C**)) or the elongation step (panel (**D**)).

To elucidate the mechanism by which SG2 prolongs lifespan in the *C. elegans* model described above, we performed in vitro studies of the Aβ aggregation process. There are three main on-pathway microscopic steps involved in the aggregation of Aβ42, i.e., primary nucleation, secondary nucleation, and elongation, with secondary nucleation being the main step that generates toxic species. The aggregation of Aβ42 was performed in unseeded and seeded assays [30,31]. Adding preformed fibrils (seeds) at low and high concentrations into the Aβ42 solution enables the quantification of the impact of SG2 on the secondary nucleation and the elongation steps, respectively. When 2% seeds are added to the solution, the primary nucleation step is bypassed, and Aβ42 aggregation proceeds through the secondary nucleation pathway, which is a process that is catalysed by the surface of the fibril. If the seed concentration is increased up to 50%, the aggregation process is dominated by elongation, with the Aβ42 monomers elongating on the fibril ends (Figure 2A). Under seeded conditions, there is no significant change after adding SG2 to the Aβ42, meaning that SG2 does not affect the secondary nucleation or elongation at the tested concentration (Figure 2C,D). However, in unseeded conditions, SG2 has a mild effect on accelerating the Aβ42 aggregation in a dose-dependent manner (Figure 2B), which indicates that SG2 slightly promotes the primary nucleation. In this case, the production of Aβ42 oligomers is expected to be only slightly affected by adding SG2, as the secondary nucleation is the main resource of generating such toxic species.

### 2.3. SG2 Activates Autophagy in a C. elegans Model of AD

**Figure 3:**
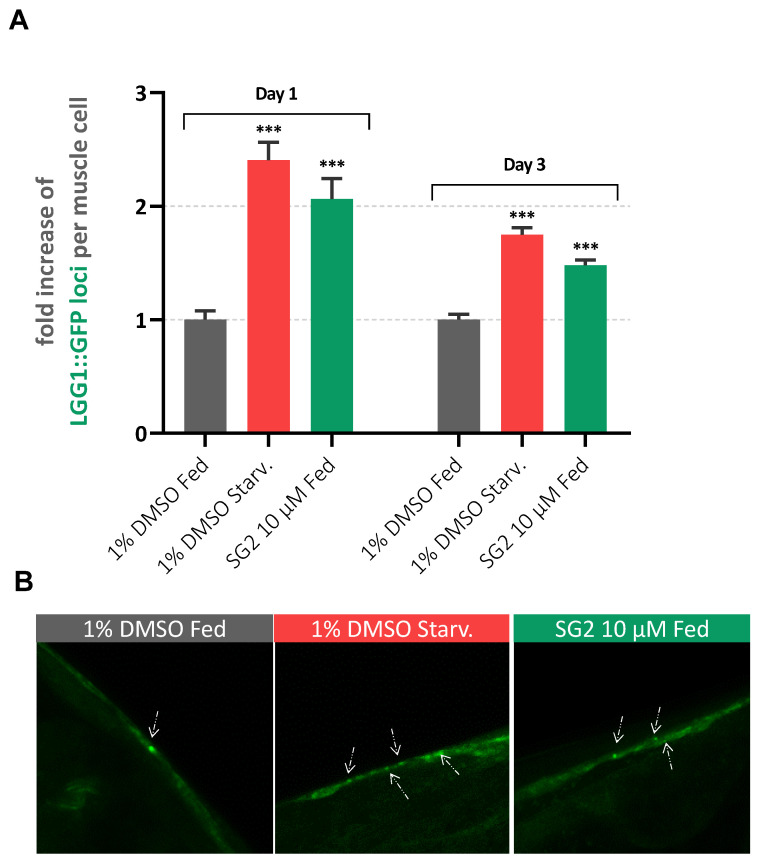
SG2 enhances autophagy in a *C. elegans* model of AD. (**A**) GFP structures were counted in 3 different conditions (Fed: 1% DMSO; Starved: 1% DMSO and 6 h of starvation; Treated: 10 µM SG2 in 1% DMSO) of approximately 25–30 worms from two different biological experiments. Bars represent average number of GFP structures normalized to control (Fed) + SEM. For statistical tests, One-Way ANOVA was used *p* < 0.001 ***. (**B**) Representative pictures of big muscles cells presenting LGG-1:GFP puncta with white arrows highlighting LGG-1::GFP structures.

Since SG2 showed to promote lifespan of the *C. elegans* model described above without interfering directly with Aβ aggregation, we wondered if this effect could be linked to the activation of autophagic pathways. We previously demonstrated that SG2 is capable of promoting a rebalance of the autophagic flux in vitro [21], which could help in the removal of misfolded proteins and neurotoxic aggregates. A generally accepted model for monitoring autophagy involves *C. elegans* strains expressing a GFP-reporter bound to LGG-1, the corresponding orthologue of human microtubule-associated proteins 1A/1B light chain 3B (LC3) [32,33]. LGG-1 plays a prominent role in autophagosome formation, as its lipidated form mediates the conjunction of adjacent membranes stimulating autophagosome membrane fusion during autophagy. Thus, a GFP reporter on this protein enables the visualization of the formation of autophagic structures in different tissues of the worms. When autophagy is induced in lgg-1::GFP strains, LGG-1 foci could be manually counted in order to evaluate autophagic promotion [33]. To confirm autophagy activation in a *C. elegans* model of AD, SG2 was administrated to a DA2123/CL2006 cross strain [34], resulting from the genetic crossing of the strain DA2123, expressing the LGG-1::GFP reporter, and CL2006, which manifest aggregation of human Aβ monomers in big muscle cells. As positive control, autophagy was induced by starving worms for 6 h [34]. As shown in Figure 3A, an increase in GFP foci was observed in SG2-treated worms at D1 and D3 following the early treatment described above for motility assays.

### 2.4. Effects of SG2 in a Cell Model of Aβ-Induced Neurotoxicity

In order to confirm the beneficial effects of SG2 against Aβ neurotoxicity in vitro, we aimed at determining whether SG2 protects U87MG human glioblastoma cells from toxic effects induced by Aβ25-35, a neurotoxic peptide commonly used in cellular models of AD [35,36]. A dose- and time-dependent reduction of cell viability was observed in U87MG cells exposed to Aβ25-35 peptide (Figure S2). Indeed, treatment of U87MG cells with increasing concentrations of Aβ25-35 (0.1, 1, 10, 25 and 50 µM) for different amounts of time (24, 48 and 72 h) revealed that exposure to 25 µM Aβ25-35 for 72 h was able to produce the most relevant reduction of U87MG cells viability (35.2%), as detected by MTT assays (Figure S2). To examine whether pre-treatment with SG2 can prevent the cytotoxic effect induced by the Aβ25-35 peptide, U87MG cells were exposed to 10 µM SG2 for 24 h, followed by treatment with 25 µM Aβ25-35 for 72 h (Figure S3A). In another set of experiments, we also assessed the ability of SG2 treatment (10 µM, 24 h) to rescue cells viability in U87MG cells previously exposed to 25 µM Aβ25-35 for 72 h (Figure S3B). Our results indicate that pre-treatment with SG2 efficiently prevent Aβ25-35 cytotoxicity in U87MG cells (Figure S3A). In U87MG cells previously exposed to 25 µM Aβ25-35 for 72 h, treatment with 10 µM SG2 for 24 h revealed to significantly rescue cell viability, even though to a lower extent as compared to the pre-treatment with SG2 (Figure S3B).

To ascertain whether the exposure of U87MG cells to 25 µM Aβ25-35 for 72 h may further reduce their autophagic activity, a gene expression analysis of autophagy-related genes was undertaken. Using a quantitative polymerase chain reaction assay (qPCR), we found that treatment with 25 µM Aβ25-35 for 72 h led to significant overexpression of the mechanistic target of rapamycin (mTOR), a master sensor of energy status and a potent inhibitor of autophagy (Figure S4A). A concomitant reduced expression of the sigma-1 receptors (σ1R) was also observed (Figure S4B), further contributing to the inhibition of autophagic activity and protein homeostasis capacity in U87MG cells [37]. In addition, we monitored the expression levels of sirtuins, since accumulating evidence has indicated that these proteins confer protection from a wide array of metabolic and age-related diseases, including obesity and neurodegeneration [38]. In particular, recent studies have also suggested that sirtuins regulate autophagy, and the cross-regulation between sirtuins and autophagy may potentially lead to effective strategies to combat aging and aging-related diseases [39]. We observed a significant down-regulation of the expression of pro-autophagy nuclear sirtuins (Figure S4C,E), namely sirtuin 1 (SIRT1) and sirtuin 6 (SIRT6), in U87MG cells incubated with 25 µM Aβ25-35 for 72 h. Notably, in the same experiments, overexpression of mitochondrial sirtuin 5 (SIRT5), a well-known inhibitor of autophagy and mitophagy [40], was also observed (Figure S4D).

The pre-treatment with SG2 (10 µM, 24 h) efficiently abolished the effects of Aβ25-35 on the expression of mTOR, σ1R, SIRT1, SIRT5 and SIRT6 (Figure S4). Notably, the increase with respect to the control cells of the expression of pro-autophagic genes, including σ1R, SIRT1 and SIRT6, appears particularly significant after pre-treatment with SG2 (Figure S4B,C,E). Consistently, in the same set of experiments, an increased expression of LC3, a specific marker of autophagy, was also observed (Figure S4F).

### 2.5. In Vitro ADME-Tox Profiling of SG2

**Figure 4:**
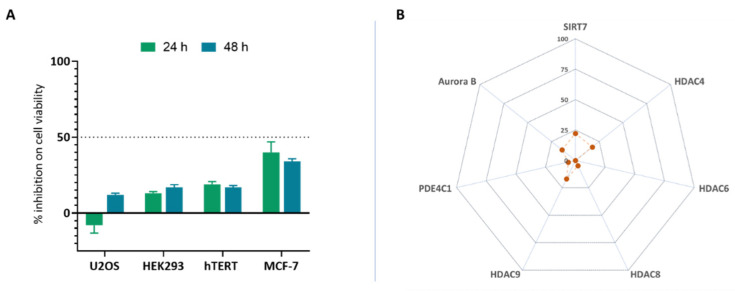
SG2 shows a safe toxicological profile in an in vitro panel. A panel of toxicological assays were performed to test (**A**) SG2 behaviour over cytotoxicity (U2-OS, HEK293, MCF-7, hTERT), (**B**) epigenetic modulation (HDAC6, HDAC8, SIRT7), and off-target liability (PDE4C1, Aurora B kinase). All assays were performed at 10 µM in triplicate. Raw data were normalized over positive and negative control and reported as percentage of inhibition. In no assay SG2 showed a percentage of inhibition higher than 40%, where in this profiling a value < 50% is generally considered acceptable.

To understand the clinical relevance of SG2 in terms of toxicological profile and in vivo stability, it was profiled in an in vitro ADME-Tox assay panel as previously described [41]. One of the first steps in ADME-Tox profiling is the assessment of the cytotoxicity of a compound. Regarding in vitro models, cytotoxic compounds significantly interfere or alter cellular attachment, morphology, growth rate or even induce cellular death [42]. Therefore, we primarily focused on understanding whether SG2 could elicit this undesired effect. U2OS (osteosarcoma), hTERT (lung fibroblast), MCF7 (human breast adenocarcinoma), HEK293 (human embryonic kidney) cell lines were selected for this study, and cytotoxicity was assessed by measuring ATP concentration which is proportional to metabolically active cells. We administrated SG2 for 24 or 48 h at a concentration of 10 µM to selected cell lines, and we did not observe any noteworthy cytotoxic effect (Figure 4A).

Another important step in assessing small molecules for progression in the drug discovery process is the determination of their ability to modulate well-known off-targets, which may induce undesirable side-effects. A small panel of assays such as phosphodiesterase (PDE), histone deacetylases (HDACs) and specific kinases, especially those involved in cell cycle regulation, could be used to profile compounds in order to reduce the costs and time when ranking compounds at an early stage. Contextually, it has been recently showed that SG2 effects could be mediated by the activation of HDAC or sirtuin enzymes and specifically, SIRT4 and SIRT6 [23]; evaluating activity of new compounds against other epigenetic enzymes could be relevant in off-target liability profiling. We selected epigenetic modulators (HDAC4, HDAC6, HDAC8, HDAC9, SIRT7) and specific kinases (Aurora B, PDE4C1) to identify off-target effects linked to SG2 [43]. In all assays SG2 tested at 10 µM showed a percentage inhibition over cell proliferation and off-target activity < 30%, with a value < 50% being considered acceptable in term of safety (Figure 4B).

### 2.6. In Vivo Toxicological Analysis of SG2 in Zebrafish (Danio Rerio)

**Figure 5:**
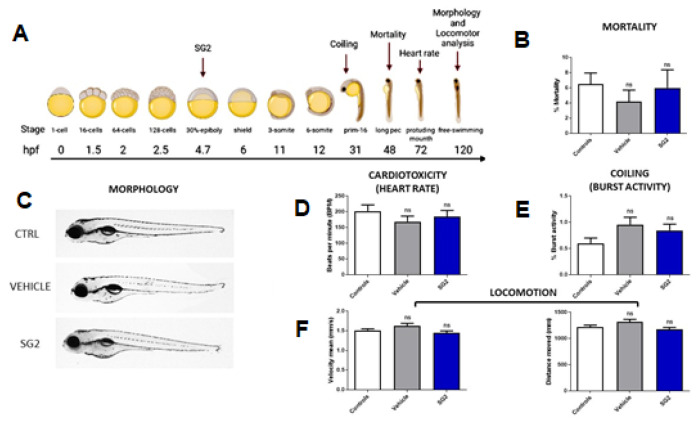
SG2 does not show toxicological effects in zebrafish. (**A**) SG2 at 5 µM was administered to zebrafish embryos at 4 h post fertilization (hpf); the experimental workflow was created with BioRender.com( accessed on 5 May, 2021). All the experiments reported show non-significant values compared to controls, proving a safe impact of SG2 in zebrafish. (**B**,**C**) Mortality rate was calculated until 48 hpf (**B**) (controls *N* = 545, vehicle *N* = 281, SG2 *N* = 224) and no morphological anomaly was observed at 5 days post fertilization (dpf) (**C**). (**D**) Cardiotoxicity was evaluated measuring heart rate at 3 dpf. (**E**,**F**) Neurological development was evaluated at 30 hpf with burst activity analysis (**E**) and at 5 dpf with locomotor measurements considering velocity and distance covered (**F**) (controls *N* = 366, vehicle *N* = 212, SG2 *N* = 246). All experiments were performed at least in triplicate. Error bars represent the SEM. For statistical tests non-parametric one-tailed Mann-Whitney rank sum test was used.

To further confirm the safety profile of SG2, we investigated its toxicological effects in vivo using a vertebrate model, zebrafish (Danio rerio), to predict a therapeutic window between efficacious and toxic exposure. Starting from the data obtained on *C. elegans* model, two concentrations of SG2 (5 and 10 µM) were tested. Since the highest concentration (10 µM) revealed a slightly toxic effect on zebrafish neurodevelopment (Figure S5), we proceeded with the toxicological analyses at 5 µM concentration. This dose revealed that SG2 does not exert any adverse effect on embryonic development, as confirmed by the mortality rate up to 2 days post fertilization (dpf), which was not different from untreated controls (Figure 5B). Moreover, the morphological (Figure 5C), cardiac (heart rate, Figure 5D) and neurological development (coiling and locomotion; Figure 5E,F) in zebrafish larvae was found to be normal, thus confirming the safety of the drug, ruling out any toxic effects on the development of other organs.

### 2.7. SG2 Treatment Improves Learning and Memory in 5XFAD Mice

**Figure 6:**
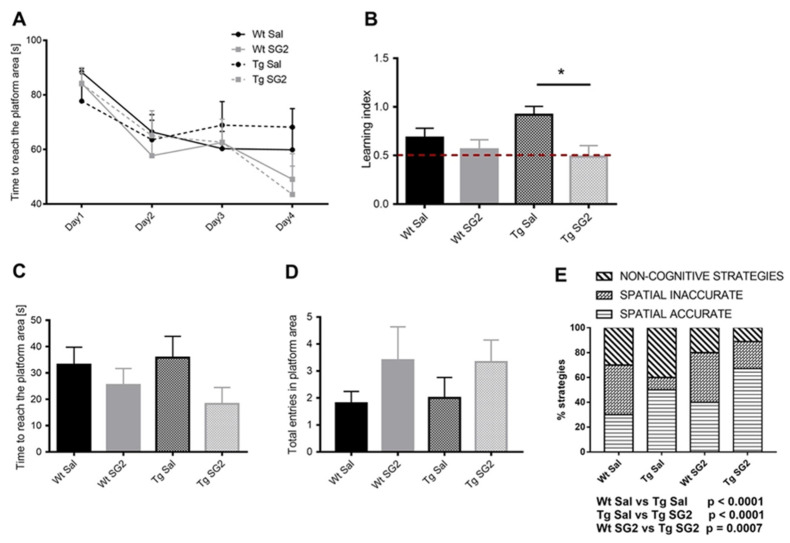
SG2 improves learning ability in the 5xFAD mouse model of AD. (**A**) Analysis of MWM learning curve showed no significant differences between experimental groups (repeated measures ANOVA, Time F (3,105) = 21.57, *p* < 0.0001, Treatment F (3,35) = 0.3141, *p* = 0.82, Time × Treatment F (9,105) = 1.553, *p* = 0.14). (**B**) Learning index analysis demonstrated an improved performance of Tg-SG2 mice compared to Tg-Sal to find the hidden platform. For statistical tests, One-Way ANOVA was used *p* < 0.02 * (**C**) The time needed to reach the platform area (**C**) and the number of entries in the target area (**D**) were not statistically different between experimental groups. (**E**) The analysis of search strategy showed an increase in spatially targeted strategies in Tg-SG2 mice compared to Tg-Sal mice (Fisher’s test). Data are shown as mean ± SEM. Experimental groups: Wt-Sal, *n* = 10; Wt-SG2, *n* = 10; Tg-Sal, *n* = 10: Tg-SG2, *n* = 9.

To assess whether SG2 treatment exerts protective effects during the early phase of AD pathology in a mouse model of AD, 5XFAD (Tg) mice were tested in the Morris Water Maze (MWM) task. Mice were trained to find a hidden platform in the MWM for four days (learning phase) and were subjected to the probe test 24 h after the last training (probe test). Even if the analysis of learning curve demonstrated no statistically significant differences between experimental groups, the learning index analysis (one way ANOVA, F (3,35) = 3.648, *p* = 0.02) showed an improved performance of Tg-SG2 mice compared to Tg-Sal mice (Figure 6B). Young mice of both genotypes do not show differences in the learning ability to find the hidden platform, since no memory deficits occur at this age. However, as reported in Figure 6A, spatially directed strategies are increased in Tg-SG2 mice compared to Tg-Sal. These results suggest that, albeit the learning ability is preserved in young Tg mice, the capability to coordinate and create navigation maps in the maze is improved by SG2 treatment. (Figure 6A).

During the probe test, that measures memory ability, even if no differences were observed either in the time to reach the platform area (one way ANOVA, F (3,35) = 3.648, *p* = 0.29) (Figure 6C) nor in the total entries in the platform area (one way ANOVA, F (3,35) = 0.9675, *p* = 0.42) (Figure 6D), the analysis of searching strategy demonstrated an increase of spatial accurate strategy of Tg-SG2 mice compared to Tg-Sal mice (Fisher’s test, *p* ≤ 0.0001) (Figure 6E). Taken together, these results suggested that SG2 treatment improves cognitive performance of 5XFAD mice at two months of age, when intracellular Aβ (iAβ) expression, extracellular Aβ (eAβ) plaque formation and neuro-inflammation are accompanied by slight behavioural deficits [44,45].

## 3. Discussion

With the FDA approval of aducanumab, both academia and industry have renewed their efforts towards the identification of therapies capable of reversing or slowing down AD progression [12]. Approximately 86% of the 126 agents currently employed in AD clinical trials are intended to be used as disease-modifying treatments (DMTs), with most of them acting through recovery of metabolism or protein homeostasis, neuroprotective effects or against amyloid. Unfortunately, only 3 DMT small molecules have progressed to Phase III clinical trials since 2020. Developing DMT small molecules for AD would facilitate an accessible, inclusive, easy administrable cure to everyone suffering from this debilitating disease.

A critical issue in the quest for AD therapies is represented by the highly complex and poorly diagnosable pathogenesis of AD [1,46]. From protein homeostasis collapse to impairment of gut-microbiota axis, there is a tangled network of dysfunctional pathways cooperating to aggravate the AD phenotype. Increasing evidence shows that polypharmacology could be an intriguing route towards this end. However, identifying DMT polypharmacological small molecules is a complex and high-risk process, which requires a good chemical starting point for drug design supported by a multidisciplinary network of in vitro and in vivo approaches. This is particularly relevant for assessing biological effects that could be beneficial in central nervous system (CNS) disorders, especially in neurodegenerative diseases. This is exemplified by triiodothyronine (T3) and tetraiodothyronine (T4) that possess many physiological actions, including ensuring CNS drug development, and neuronal differentiation and migration. Dysfunction of TH signalling can lead to cognitive and mnemonic problems with impairment of motor and language abilities. Recently, THs and analogues thereof have been shown to preserve mitochondrial homeostasis by inducing autophagy [47,48]. However, the molecular mechanisms underlying their pleiotropic effects remain unclear. Prompted by their interesting profile and relatively explored chemistry, over the last five years we have focused on the development of safe synthetic analogues of THs and their metabolites. Among our library of small molecules, we identified SG2 as a target-orphan synthetic analogue of thyronamine with a promising in vitro polypharmacological profile. We report herein that the use of a combination of in vitro and in vivo models revealed that SG2 has a potential to treat AD.

We observed a significant effect of SG2 at 10 µM in a *C. elegans* model of AD, both as preventive and therapeutic strategies. Given that the main pathological hallmark of this AD model is the expression of Aβ1-42 aggregates in vivo, we evaluated the effect of SG2 on the Aβ aggregation process. As we did not observe a significant effect of SG2 in slowing down or inhibiting any step of the Aβ aggregation process, we considered whether the positive effect observed in the AD model could be elicited by other biological effects associated with SG2 administration. Following the recent evidence that SG2 promotes autophagy in vitro, we investigated SG2 using a double transgenic *C. elegans* strain expressing both Aβ1-42 and GFP-tagged LGG-1 (the worm orthologue of human LC3). By performing confocal microscopy studies, we observed an increase in autophagic structures in *C. elegans* tissues where Aβ expression is localized. This result suggests that a potential mechanism of action of this compound could involve the activation of the autophagy-lysosomal pathway. To support this potential mechanism, we generated a human cell model of Aβ toxicity and assessed the effects of SG2 administration, which confirmed the efficacy of SG2 against Aβ toxicity; further gene expression analysis showed an increase in genes closely related to the autophagic process. These results indicated that SG2 could represent a valuable pharmaceutical tool against AD. Before moving to the testing in a mouse model of AD, SG2 was shown to have a safe profile in an in vitro ADME-Tox assay panel as well as in a zebrafish vertebrate model, that also helped in the prediction of a therapeutic window between efficacious and toxic exposure. We then tested SG2 in young 5XFAD mice, a severe mouse model of AD. Our preliminary results demonstrated that spatial learning and memory are significantly, though modestly, improved in 2-months 5XFAD mice treated with SG2 compared to their littermates treated with vehicle. Tg-SG2-treated mice showed indeed a marked improvement in the performance through the test sessions (learning index) and an improved capability to use spatially precise trajectories to find the hidden platform. The exact mechanism by which SG2 exerts neuroprotection in vivo is yet unexplored; moreover, further studies will assess whether SG2 treatment during the early phase of AD is also able to slow down AD progression in older mice and to reduce Aβ-induced neuro-inflammation.

## 4. Materials and Methods

### 4.1. Aβ Aggregation

Aβ42(M1-42) was expressed in E. coli BL21 Gold (DE3) with a methionine at the N-terminus (elsewhere referred to as Aβ42) and the inclusion body was extracted and purified using ion-exchange (IEX) and size-exclusion chromatography (SEC). The target protein was eluted in buffer with 125 mM NaCl during the IEX from the DE23 anion exchange resin and followed by two rounds of SEC using Superdex75 increase column (GE Healthcare). The protein was purified and diluted to the required concentration in 20 mM sodium phosphate buffer at pH 8.0 with 0.2 mM of EDTA. Preformed fibrils (seeds) were prepared by aggregating 5 µM monomeric at 37 °C and diluted to the needed concentration. SG2 was dissolved in MQ water at a stock concentration of 5 mM. The molecule was added to Aβ42 to reach a final concentration of 5 µM to 20 µM. 20 µM thioflavin T (ThT) was added to the solution to serve as a fluorescence probe for the aggregation. The kinetics of Aβ42 aggregation was recorded in the Fluostar plate reader (BMG Labtech) with an excitation wavelength at 440 nm and emission wavelength at 480 nm.

### 4.2. C. elegans Experiments

#### 4.2.1. Media

Standard conditions were used for the propagation of *C. elegans* [49]. Briefly, the animals were synchronized by hypochlorite bleaching, hatched overnight in M9 buffer (3 g/L KH_2_PO_4_, 6 g/L Na_2_HPO_4_, 5 g/L NaCl, 1 M MgSO_4_), and subsequently cultured at 20 °C on nematode growth medium (NGM; 1mM CaCl_2_, 1 mM MgSO_4_, 5 µg/mL cholesterol, 250 M KH_2_PO_4_ pH 6, 17 g/L agar, 3 g/L NaCl, 7.5 g/L casein) on plates previously seeded with the *E. coli* strain OP50. In order to prepare these plates, saturated cultures of OP50 were grown by inoculating 50 mL of LB medium (10 g/L tryptone, 10 gL^−1^ NaCl, 5 gL^−1^ yeast extract) with OP50 and incubating the culture for 16 h at 37 °C. The NGM plates were seeded with bacteria by adding 350 µL of saturated OP50 to each plate and leaving the plates at 20 °C for 2–3 days. On day 3 after synchronization, the animals were placed on NGM plates containing 5-fluoro-2′deoxy-uridine (FUDR) (75 µM, unless stated otherwise) to inhibit the growth of offspring.

#### 4.2.2. Strains

Following strains were used in this study: Bristol N2, wildtype (wt); GMC101, dvIs100 [unc-54p::Aβ1-42::unc-54 3′-UTR + mtl-2p::GFP]; DA2123/CL2006 [lgg-1p::GFP::lgg-1 + unc-54p::Aβ1-42::unc-54 3′-UTR + rol-6 The gene unc-54p::Aβ-1-42 expresses the full-length human Aβ1-42 peptide which self-aggregates in vivo in body wall muscle cells. L4 or young adults carrying this gene undergo paralysis when temperature is shifted from 20 °C to 25 °C. lgg-1p::GFP::lgg-1 produces constitutive expression of GFP linked to lgg-1, which is homologue for LC3 in *C. elegans*. In standard conditions lgg-1::GFP is diffuse in the cytosol, but when autophagy is induced (e.g., by starvation) it associates with nascent autophagosome membranes and it’s punctuate in structure. Therefore, quantification of green puncta is widely used to monitor autophagy induction in several *C. elegans* tissues [50].

#### 4.2.3. Compound Administration

At D0 or D4 of adulthood, 2.2 mL aliquots of compounds dissolved in DMSO at different concentrations were spotted onto the FUDR plates as previously described [28,29]. The plates were then placed in a sterile laminar flow hood at RT to dry. For the experiments, worms were transferred onto the compound-seeded plates directly at D0 or D4 and they were exposed to SG2 for their whole lifespan.

#### 4.2.4. Automated C. elegans Motility Assays

All *C. elegans* populations were cultured at 20 °C and developmentally synchronized from a 4 h egglay. At L4 stage individuals were transferred to FUDR plates, and the temperature was raised to 24 °C to induce Aβ aggregation and worm paralysis. For late treatment worms were transferred on FUDR plates at D4. At different ages, the animals were washed off the plates with M9 buffer and spread over an OP50 unseeded 9 cm plate, after which their movements were recorded at 20 fps using a recently developed microscopic procedure for 1 min [28]. Up to 1200 animals were counted in each experiment unless otherwise stated. One experiment that is representative of the three measured in each series of experiments is shown, and videos were analyzed using a custom-made tracking code [28].

#### 4.2.5. Confocal Microscopy

The DA2123/CL2006 strain was used to assess autophagy induction in *C. elegans*, after treatment with SG2. At Day 1 or Day 3, 30 worms from each condition (10 µM SG2; 1% DMSO, fed; 1% DMSO, starved) were scooped up avoiding bacteria and transferred onto a 2% agarose pad located on a glass slide and containing a levamisole drop (3.5 µL). Then, worms were pressed under a glass slide avoiding air bubble formation and imaged immediately to prevent loss of fluorescence signal. Confocal images were acquired as Z-stack images (from 10 to 30 slices per image) using a Nikon A1R confocal microscope at 20× or 40× magnification, adjusting brightness and contrast equally between different conditions. Brightness and contrast of images were adjusted equally between the different conditions. Z-projection was obtained from acquired images using Fiji, and the number of lgg-1::GFP positive loci in each Z-projection manually counted from two independent experiments.

### 4.3. Zebrafish Experiments

#### 4.3.1. Zebrafish Husbandry

Experiments were carried out using the wild-type AB strain. Adults were housed in tanks at a density of no more than five zebrafish per litre at a constant temperature of 28 °C on a 14 h light/10 h dark cycle. Zebrafish eggs and embryos were collected and raised at 28.5 °C in E3 medium using established procedures and staged in hours post fertilization (hpf) or days post fertilization (dpf) [51]. All experiments were conducted in accordance with the European Union (EU) Directive 2010/63/EU on the protection of animals used for scientific purposes, and under the supervision of the Institutional Animal Care and Use Committee of the IRCCS Fondazione Stella Maris, and complied with the 3R principles [52].

#### 4.3.2. Toxicological Analyses on Zebrafish Embryos and Larvae

Stock solution of SG2 was prepared in DMSO and diluted in egg water to the final administered concentrations. Normally developing embryos were selected under a stereomicroscope at 4 hpf, and randomly placed in 60 mm × 15 mm petri dishes at a density of 50 per dish, containing the SG2 molecule diluted in egg water, or 0.1% DMSO diluted in egg water, for the vehicle group, or only egg water for the control (untreated) group. To select the appropriate working dilution a preliminary dose-dependence test was performed starting from the doses already tested on *C. elegans*. For each experiment, at least three independent assays were performed. Mortality was calculated comparing death rates between all experimental groups at 24 and 48 hpf. To evaluate the effect on embryo development, a morphological analysis was performed on live zebrafish larvae at 5 dpf using a glass depression slides with 3% agarose. Images were obtained using a Leica M205FA stereomicroscope (Leica Microsystem, Wetzlar, Germany). The cardiotoxicity effect of the SG2 compound was verified, measuring the heartbeat in zebrafish larvae at 72 hpf, through a video recording of 15 s at 30 frame/sec for at least 15 larvae per each experimental group in triplicate. Data analysis of the heart rate was performed using Danioscope software (Noldus©, Wageningen, The Netherlands). Behavioral analysis was evaluated in early stages, such as 30 hpf, performing the coiling assay where the number of complete body contractions of each embryo (*n* = 30 for each experiment) made in a 30 s period was counted using Danioscope software (Noldus©, Wageningen, The Netherlands). We also analyzed locomotion at later stages, in 120 hpf larvae in each experimental group. The larvae were transferred into 96-well plates containing 200 μL of egg water per well. Each plate was placed in the DanioVision^®^ device (Noldus©) and the larval activity was recorded for 30 min and analysed using EthoVision XT^®^ software version 12 (Noldus©, Wageningen, The Netherlands). Statistical analysis was performed on three or more independent experiments. We performed the statistical analysis using GraphPad Prism 6 software. In animal studies, all data were analysed using post hoc comparisons, performed by Tukey HSD. The significance between groups was determined using the non-parametric one-tailed Mann–Whitney rank sum test, as indicated in each figure legend. Statistical significance is reported as: * *p* ≤ 0.05, ** *p* ≤ 0.01, *** *p* ≤ 0.001, or **** *p* ≤ 0.0001.

### 4.4. Mice Experiments

#### 4.4.1. Mouse Model

For this study, male and female two-months old 5xFAD heterozygous mice and their wild-type (Wt) littermates were used. The 5xFAD transgenic mouse model of AD [44,45] co-overexpresses a triple-mutant human amyloid precursor protein (APP) (Swedish mutation: K670N, M671L; Florida mutation: I716V; London mutation: V717I) and a double-mutant human presenilin 1 (PS1) (M146L and L286V mutations) under the transcriptional control of the neuron-specific Thy-1 promotor. Progenitors with no retinal degeneration allele Pde6brd1 were purchased from Jackson Laboratories, Bar Harbor, and progenies were obtained by crossing hemizygous 5xFAD mice with B6SJL/J breeders. Mice were kept in conditioned rooms with stable temperature (21 °C) and humidity (60%), on a light/dark cycle of 12 h. Food and water were available ad libitum and body weight was recorded throughout the entire observation period. All animal procedures were approved by the Committee on Animal Health and Care of the University of Modena and Reggio Emilia (protocol number: n°619/2020-PR, on 24 June 2020) and conducted in accordance with National Institutes of Health guidelines.

#### 4.4.2. Drugs and Procedures

Mice received a daily subcutaneous injection of SG2 or vehicle (saline) for 30 days at a dose of 1.3 mg/kg. The SG2 dose for in vivo study was selected based on in vitro experiments. Forty mice were randomly divided into 4 groups and received vehicle (saline, Sal) or SG2: Wt Sal, Wt SG2, Tg Sal and Tg SG2.

#### 4.4.3. Behavioural Analysis

To assess spatial learning and memory mice underwent the Morris water maze (MWM) test on Day 25 of treatment, as previously described [53,54]. During the learning phase, mice were placed in a circular white pool with a diameter of 120 cm filled with water at 22 ± 1 °C (made opaque by adding white non-toxic paint), and allowed to swim for 90 s or until they found the location of a hidden circular platform with 11 cm diameter, mice were trained with 4 trials per day (starting from a different quadrant with different visual cues at each trial) for 4 days with 60 min inter-trial interval, For each trial, the time to reach the platform and mean speed were (learning curve). As a learning index, we calculated (time to target at 4th day/time to target at the first day). On the 5th day, to assess memory retention, the platform was removed, and the animals were allowed to swim for 60 s (probe test), and the latency to reach the platform area, total entries in the platform area and the % of time spent in target quadrant were evaluated [55]. The analysis of target search strategy was carried out according to current literature [56]. Briefly, spatial accurate strategy and non-spatial strategy were distinguished considering individual swim paths during probe test.

The behavioural test was performed by an operator unaware of the treatment to avoid bias. Animal behaviour was conducted in a sound-proof room, recorded and automatically analyzed with ANY-maze Video Tracking system (Stoelting). The target search strategy was manually scored, independently, by two operators unaware of the treatment.

#### 4.4.4. Statistical Analysis

All data are shown as mean ± standard error of the mean (SEM) and were analysed by one-way analysis of variance (ANOVA), two-way repeated measures analysis of variance (ANOVA) followed by the Bonferroni‘s test, and Fisher’s test using the statistical package SPSS (version 26). Differences were considered significant with *p*-value *p* < 0.05, with *p* < 0.05 *; *p* < 0.01 **; *p* < 0.001 ***.

## 5. Conclusions

To conclude, in this work we investigated the potential role of a target-orphan tyroxine derivative, namely SG2, that was previously developed and studied by our group. Particularly, using a multidisciplinary approach combining in vitro assays, ADME-Tox profiling, and in vivo studies, we investigated its therapeutic effect for treatment of AD. From the work reported herein, SG2 has emerged as a polypharmacological and quite safe agent for the treatment of AD. Indeed, it showed to significantly promote lifespan in AD *C. elegans* model through activation of the autophagy-lysosomal pathway. Similarly, we demonstrated that SG2 has a safe toxicological profile both in vitro and in vivo at 5 µM, a concentration that exerts beneficial effects in *C. elegans*. Finally, we further confirmed the therapeutic potential of SG2 in transgenic mice models of AD, demonstrating that it promotes cognitive and learning effects. Our results indicate that thyroid hormone derivative SG2 may serve as promising molecule in an effort to treat AD as well as other neurodegenerative diseases characterized by accumulation of abnormal proteins and autophagic dysfunction. Given that the molecular target(s) of SG2 is still unknown, further studies are required to clarify the biological mechanism(s) that could explain its interesting polypharmacology. To this end, we are currently engaged in a medicinal chemistry program with target deconvolution and hit-to-lead optimization studies.

## Data Availability

Data is contained within the article and Supplementary Files.

## Supplementary Materials

The following are available online at https://www.mdpi.com/article/10.3390/ph14121330/s1, Figure S1. Chemical Structure of SG2, Figure S2. Analysis of Aβ25-35 cytotoxicity in U87MG cells. (A) U87MG cells were pre-treated with SG2 compound (10 µM) for 24 h. Following incubation time, cells were incubated with of Aβ25-35 oligomers (25 µM) for 72 h. (B) U87MG cells were exposed to of Aβ25-35 oligomers (25 µM) for 72 h and then post-treated with SG2 compound (10 µM) for 24 h. Following incubation time MTT cell viability assay was carried out. Each experiment was performed in triplicate and values represent the mean ± SEM. Statistical analysis was performed by ordinary one-way ANOVA followed by Tukey’s test; * *p* < 0.05; ** *p* < 0.01, Figure S3. Effects of SG2 treatment on amyloid-induced cytotoxicity. U87MG cells were treated with increasing concentrations of Aβ25-35 (0.1, 1, 10, 25 and 50 µM) for different amount of time: 24 h (A), 48 h (B) and 72 h (C). Following incubation time, MTT cell viability assay was carried out. Each experiment was performed in triplicate and values represent the mean ± SEM. Statistical analysis was performed by ordinary one-way ANOVA followed by Tukey’s test; * *p* < 0.05; ** *p* < 0.01; *** *p* < 0.001, Figure S4. Real time quantification of autophagy related genes in U87MG cells exposed to pre-treatment with SG2 (10 µM; 24 h) followed by incubation with 25 µM Aβ25-35 for 72 h. Each experiment was performed in triplicate and values represent the mean ± SEM. Statistical analysis was performed by ordinary one-way ANOVA followed by Tukey’s test; * *p* < 0.05; ** *p* < 0.01; *** *p* < 0.001, Figure S5. SG2 10µM effects in zebrafish. SG2 was administered at 10 µM to the zebrafish at 4 hpf. This dose revealed a slightly toxic effect on zebrafish neurodevelopment. Mortality rate was calculated until 48 hpf (A) (controls N = 220, vehicle N = 143, SG2 N = 141) and no morphological anomaly was observed at 5 dpf (B). Cardiotoxicity was evaluated measuring heart rate at 3 dpf (C). Neurological development was evaluated at 30 hpf with burst activity analysis (D) and at 5 dpf with locomotor measurements considering velocity and distance covered (E) (controls N = 345, vehicle N = 131, SG2 N = 249). All experiments were performed at least in triplicate. Error Bars represent Standard Error on the Mean (SEM). For statistical tests non-parametric one-tailed Mann-Whitney rank sum test was used. * *p* ≤ 0.05, ** *p* ≤ 0.01, *** *p* ≤ 0.001, or **** *p* ≤ 0.0001. Only significant data are reported in all the graphs, Table S1: Primer sequences used in real time PCR experiments. Material and methods for in vitro studies (cell viability assay, gene expression analysis, cell viability assays for ADME-Tox studies, HDAC, SIRT7, PDE4C1, and Aurora B inhibition assays).
