# Supplementary material for: Identification of a Thyroid Hormone Derivative as a Pleiotropic Agent for the Treatment of Alzheimer’s Disease
Source: Pharmaceuticals (Basel). 2021 Dec 19;14(12):1330. doi: 10.3390/ph14121330 (PMC8704018; doi:10.3390/ph14121330)
Supplement: Supplementary file 1 [file pharmaceuticals-14-01330-s001.zip › pharmaceuticals-1496894-supplementary.pdf]

Supporting Information for

Identification of a thyroid hormone derivative as a pleiotropic agent for the treatment of Alzheimer's disease

Massimiliano Runfola [1], Michele Perni [2], Xiaoting Yang [2], Maria Marchese [3], Andrea Bacci [1], Serena Mero [3], Filippo M. Santorelli [3], Beatrice Polini [4], Grazia Chiellini [4], Daniela Giuliani [5], Antonietta Vilella [5], Martina Bodria [5], Eleonora Daini [5], Eleonora Vandini [5], Simon Rudge [6], Sheraz Gul [7,8], Michael OJ Wakelam [6], Michele Vendruscolo [2]* and Simona Rapposelli [1,9] *

1 *Department of Pharmacy, University of Pisa, Via Bonanno 6, 56126 Pisa, Italy*
2 *Centre for Misfolding Diseases, Department of Chemistry, University of Cambridge, Cambridge CB2 1EW, United Kingdom*
3 *Molecular Medicine, IRCCS Stella Maris, Via dei Giacinti 2, 56128 Calambrone (Pisa), Italy*
4 *Department of Pathology, University of Pisa, Via Savi 10, Pisa, 56126, Italy*
5 *Department of Biomedical, Metabolic and Neural Sciences, University of Modena and Reggio Emilia, Via G. Campi 287, 41125 Modena, Italy*
6 *The Babraham Institute, Babraham Research Campus, Cambridge, CB22 3AT, United Kingdom*
7 *Fraunhofer Institute for Translational Medicine and Pharmacology ITMP, Schnackenburgallee 114, 22525 Hamburg, Germany*
8 *Fraunhofer Cluster of Excellence Immune-Mediated Diseases CIMD, Hamburg Site, Schnackenburgallee 114, 22525, Hamburg, GermanyCISUP, Center for Instrument Sharing, University of Pisa, 56126 Pisa, Italy*
9 *CISUP, Center for Instrument Sharing, University of Pisa, 56126 Pisa, Italy*

*** Correspondence**: S.R: simona.rapposelli@unipi.it; +39 050 2219528; M.V.: mv245@cam.ac.uk

Summary

In vitro Studies. Material and Methods **3**
Cell lines and reagents 3
Cell viability assay 3
Gene expression analysis 3
Statistical Analysis 3
Cell viability assays for ADME-Tox studies 3
HDAC inhibition assays 4
SIRT7 inhibition assays 4
PDE4C1 inhibition assays 4
Aurora B inhibition assays 5

Supplementary Figures and Tables **6**
Figure S1. Chemical Structure of SG2 6
Figure S2. Effects of SG2 treatment on amyloid-induced cytotoxicity 7
Figure S3. Analysis of Aβ25-35 cytotoxicity in U87MG cells 8
Figure S4. Real time quantification of autophagy related genes in U87MG cells exposed to pre-treatment with SG2 (10 μM; 24 h) followed by incubation with 25 μM Aβ25-35 for 72 h 9
Figure S5. SG2 10μM effects in zebrafish 10
Table S1. Primer sequences used in real time PCR experiments 11

In vitro Studies. Material and Methods

Cell lines and reagents. The U87MG human glioblastoma cell line was obtained from American Type Culture Collection (ATCC, Manassas, VA, USA) and grown in DMEM High Glucose medium (Sigma-Aldrich S.r.l., Milan, Italy) supplemented with 10% fetal bovine serum (FBS), 50 IU/mL penicillin, and 100 µg/ml streptomycin (Sigma-Aldrich Srl) at 37 °C in a humidified atmosphere with 5% CO_2; the medium was renewed three times per week. The fragment 25-35 of the amyloid β peptide ($Aβ_{25-35}$) (Sigma-Aldrich Srl, Milan, Italy) was dissolved in deionized water and stocked in various aliquots at –20°C.

Cell viability assay. U87MG cells were seeded in 96-well plates at a density of $5x10^4$ cells/well and cultured for 24 h. The cells were treated with Aβ and/or SG2 at the indicated concentrations for the specified times. After treatment, the cell viability was measured by performing MTT (3-(4,5-dimethylthiazol-2-yl)-2,5-diphenyl-tetrazolium bromide) assay (Sigma-Aldrich S.r.l., Milan, Italy). Briefly, 10 µL of the MTT solution (5 mg/mL) were added to each well and incubated for 4 h at 37 °C. Then, 25 µL of supernatant were removed and 75 µL of DMSO were added into each well. The absorbance was measured at 570 nm with a microplate reader (Bio-Rad model 550 microplate reader, Bio-Rad Molecular Bioscience Group, Hercules, USA). The data represent the mean of three independent experiments.

Gene expression analysis. Cells were cultured at a density of $3×10^5$ cells/well in a 6-well plate in a final volume of 1.5 ml/well. Twenty-four hours (h) after seeding, the cells were treated with Aβ and SG2 for 24, 48, and 72 h. Dilutions of test compounds were obtained by a stock solution (50 mM in DMSO). Total RNA was extracted from U87MG cells using the Direct-zol RNA MiniPrep (Zymo Research, Irvine, CA, USA) including the on-column DNaseI treatment, following manufacturer instructions. RNA concentration and purity were determined by Nanodrop-1000 spectrophotometer (Thermo Fisher Scientific, Wilmington, DE, USA). After the retro-transcription step performed by iScriptTM gDNA Clear cDNA Synthesis Kit (Bio-Rad, Milano, Italy), the relative quantities of cDNA samples were analyzed by real-time PCR experiments using SsoAdvanced Universal SYBR Green Supermix on CFX-Connect Real-Time (RT)-PCR Detection System instrument (Bio-Rad, Milano, Italy). The PCR cycle program consisted of an initial 30 s denaturation at 95°C followed by 40 cycles of 5 s denaturation at 95°C and 15 sec annealing/extension at 60°C. A final melting protocol with ramping from 65°C to 95°C with 0.5°C increments of 5 s was performed for verification of amplicon specificity and primer dimer formation. Negative control of retro-transcription was performed to exclude any interference from residual genomic DNA contamination. Sequences of the primers for RT-PCR are reported in **Table S1**.

Statistical Analysis. All data are reported as mean ± SEM. Statistical analysis was performed by one-way analysis of variance (ANOVA), followed by Tukey's multiple comparison test. The threshold of statistical significance was set at $P <0.05$. Data analysis was performed by Graph-Pad Prism 8.4 statistical program (GraphPad Software Inc., San Diego, CA, USA).

Cell viability assays for ADME-Tox studies. Osteosarcoma cell lines (U2-OS), immortalized human cell lines (hTERT), human breast adenocarcinoma cell line (MCF7), and human embryonic kidney cell lines (HEK293) were grown on surface-modified T175 cell culture flasks in Dulbecco's Modified Eagle Medium (DMEM) with 10% fetal bovine serum (FBS), 5% of L-glutamic acid, 5% streptomycin, and 5% penicillin G. At about 80%

confluency, cells were washed, trypsinized, resuspended, and counted in DMEM before seeding at 4000 cells/well (in triplicate) into white 384-well microtiter plates (20 µL) containing 20 nL/well of SG2 (10 µM final concentration, 0.1% of DMSO), negative control Valinomycin (10 µM final concentration, 0.1% of DMSO), or DMSO as positive control, previously dispensed using an Echo 550 Liquid Handler. Plates were then and incubated at 37°C in the presence of 5% CO_2. At 24 h or 48 h after cell were seeded, 20 µL/well of CTG detection mix from CellTiter-Glo Assay Kit (Promega Corp, Madison, USA) were added, and plates were read using an EnVision Multilabel 2103 Reader after a 10 min incubation in the dark. Raw data were normalized to percentage of cell growth by using the corresponding NC containing only 0.1% v/v DMSO. The luminescence signal of each sample (S) was converted into percentage of cell growth compared with the average signal of NC. The following formula was used: % effect = (S – PC)/NC x 100.

HDAC inhibition assays. Inhibition of HDAC enzymes (in triplicate) was determined using the homogeneous, single-addition, bioluminogenic HDAC-Glo I/II assay (Promega Corp, Madison, USA). Human recombinant HDAC enzymes were purchased from BPS Bioscience (San Diego, CA), and standard inhibitor trichostatin A (Sigma-Aldrich) was dissolved to a yield stock solution in 100% v/v DMSO and stored at −20°C. Plate handling was performed using an Echo 550 Liquid Handler and luminescence measurements taken using a 2300 EnSpire Multilabel reader. SG2 and positive control (trichostatin A with final concentration of 10 µM and 0.1% v/v DMSO) and high control (final 0.1% v/v DMSO) were added into the 384-well plates (10 nL/well; 0.1% v/v DMSO) using the Echo 550 Liquid Handler. The HDAC-Glo I/II assay reagent was prepared according to kit manual and 10 µL of it were added to each well. The microtiter plates were mixed briefly by orbital shaking (500–700 rpm), and luminescence was measured at steady-state signal: background, which was achieved after 20 min.

SIRT7 inhibition assays. The determination of the inhibitory activity of SG2 on SIRT7 was determined using the SIRT-Glo assay kit (Promega Corp., Madison, USA). Human recombinant SIRT7 was purchased from BPS Bioscience (San Diego, CA). Plate handling was performed using an Echo 550 Liquid Handler and luminescence measurements taken using a 2300 EnSpire Multilabel reader. SG2 and DMSO for controls were added into the 384-well plates (10 nL/well; 0.1% v/v DMSO) using the Echo 550 Liquid Handler. SIRT-Glo reagent was prepared as described in the manual kit and added to each well after 20 min of incubation (10 µL/well). The microtiter plates were mixed briefly by orbital shaking (500–700 rpm), and luminescence was measured at steady-state signal: background, which was achieved after 30 min.

PDE4C1 inhibition assays. The inhibitory effect of SG2 on PDE4C1, a protein necessary to assure normal cell growth, was determined (in triplicate) using the PDE4C1 assay kit provided by BPS Bioscience (San Diego, CA). SG2 (10 nL, in assay concentration of 10 µM and 0.1% of DMSO) and DMSO as control were dispensed into a black 384-well no-binding low-volume plate. Substrate and enzyme solution were prepared and 2.5 µL of each mix were dispensed in each well, and the plate was gently mixed and incubated for 1h at RT at repair from light. Then, binding agent solution were prepared according to kit protocol, and 10 µL of this solution were added to each well. The plate was then sealed, centrifuged and incubated for 20 min at RT with slow shaking. Fluorescence polarization measurement were performed on a EnVision Multilabel 2103 Reader. Each signal

was normalized to positive (100% inhibition, no substrate) and to negative control (0% inhibition, DMSO control)

Aurora B inhibition assays. The inhibitory effect of SG2 on Aurora B kinase was determined (in triplicate) using the Kinase-Glo® Luminescent Kinase Assay Platform (Promega Corp.). 5 nL of 10 mM stock solution of test compounds and 5 nL of DMSO as control were dispensed to each well of a 384-well plate (final concentration 10 μM; final concentration of DMSO 0.1%) using an Echo 550 Liquid Handler. An enzyme master mix containing 1× buffer, 50 μM DTT, and 300 nM Aurora B (all reagents provided in the kit) was prepared and 2.5 μL of solution were added to each well. 2.5 μL of reaction mix containing 1× buffer, 3.33 μM adenosine triphosphate (ATP), and 7.5 ng/μL (18.75 ng/well) myelin basic protein (MBP) as substrate (buffer and MBP were provided in the Aurora B Kinase Enzyme System; ultrapure ATP was acquired by Sigma Aldrich) were added to each well in order to start reaction. Plate was then sealed using thermowell sealing taper, briefly mixed, and incubated for 30 min at RT. Following this, 5 μL of Kinase-Glo® Reagent were added to each well, the plate was then mixed and incubated at RT for 20 min, and luminescence measurements were read using a 2003 EnSpire Multilabel reader. The luminescence signal of each sample (S) was converted into percentage of AuroraB inhibition and compared with the average signal of no substrate (100% inhibition).

Supplementary Figures and Tables

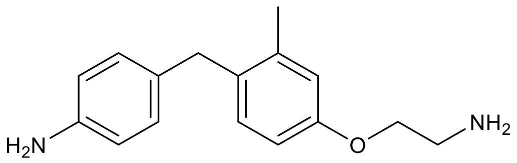

SG2

Chemical Formula: $C_{16}H_{20}N_2O$
Exact Mass: 256,16
Molecular Weight: 256,35

Figure S1. Chemical Structure of SG2.

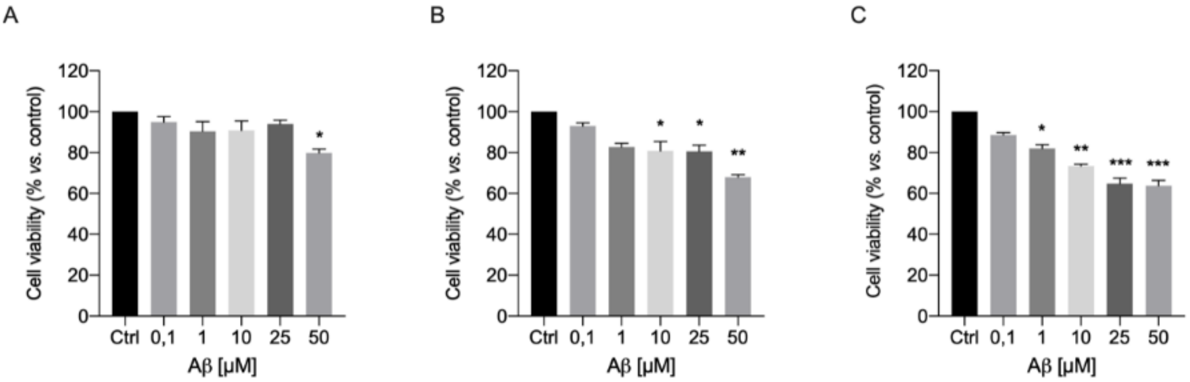

Figure S2. Effects of SG2 treatment on amyloid-induced cytotoxicity. U87MG cells were treated with increasing concentrations of Aβ25-35 (0.1, 1 10, 25 and 50 μM) for different amount of time: 24 h (**A**), 48 h (**B**) and 72 h (**C**). Following incubation time, MTT cell viability assay was carried out. Each experiment was performed in triplicate and values represent the mean ± SEM. Statistical analysis was performed by ordinary one-way ANOVA followed by Tukey's test; *p<0.05; **p<0.01; ***p<0.001.

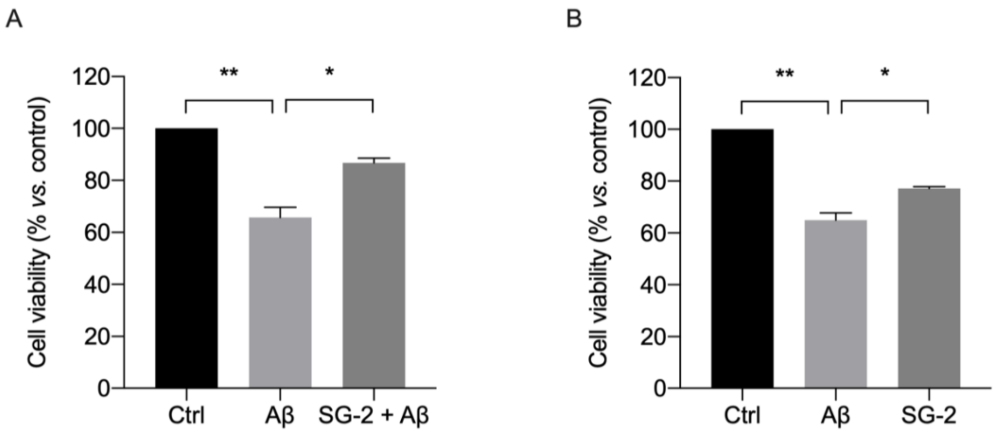

Figure S3. Analysis of Aβ25-35 cytotoxicity in U87MG cells. (**A**) U87MG cells were pre-treated with SG2 compound (10 µM) for 24 h. Following incubation time, cells were incubated with of Aβ25-35 oligomers (25 µM) for 72 h. (**B**) U87MG cells were exposed to of $A\beta_{25-35}$ oligomers (25 µM) for 72 h and then post-treated with SG2 compound (10 µM) for 24 h. Following incubation time MTT cell viability assay was carried out. Each experiment was performed in triplicate and values represent the mean ± SEM. Statistical analysis was performed by ordinary one-way ANOVA followed by Tukey's test; *p<0.05; **p<0.01.

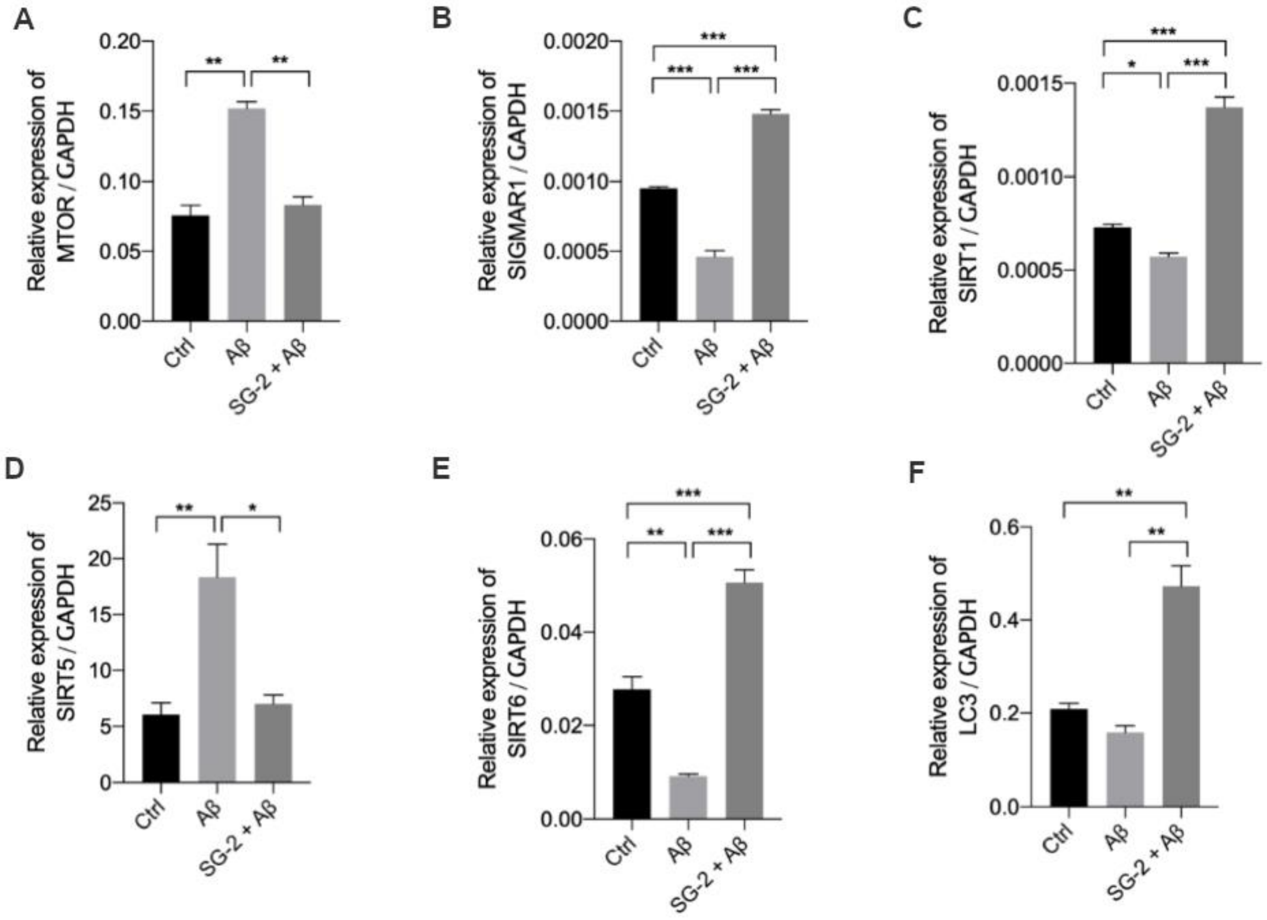

Figure S4. Real time quantification of autophagy related genes in U87MG cells exposed to pre-treatment with SG2 (10 µM; 24 h) followed by incubation with 25 µM Aβ25-35 for 72 h. Results are reported as relative expression against reference gene (GADPH) of autophagy related genes: MTOR (**A**), SIGMAR1 (**B)**, SIRT1 (**C**), SIRT5 (**D**), SIRT6 (**E**), LC3 (**F**). Each experiment was performed in triplicate and values represent the mean ± SEM. Statistical analysis was performed by ordinary one-way ANOVA followed by Tukey's test; *p<0.05; **p<0.01; ***p<0.001

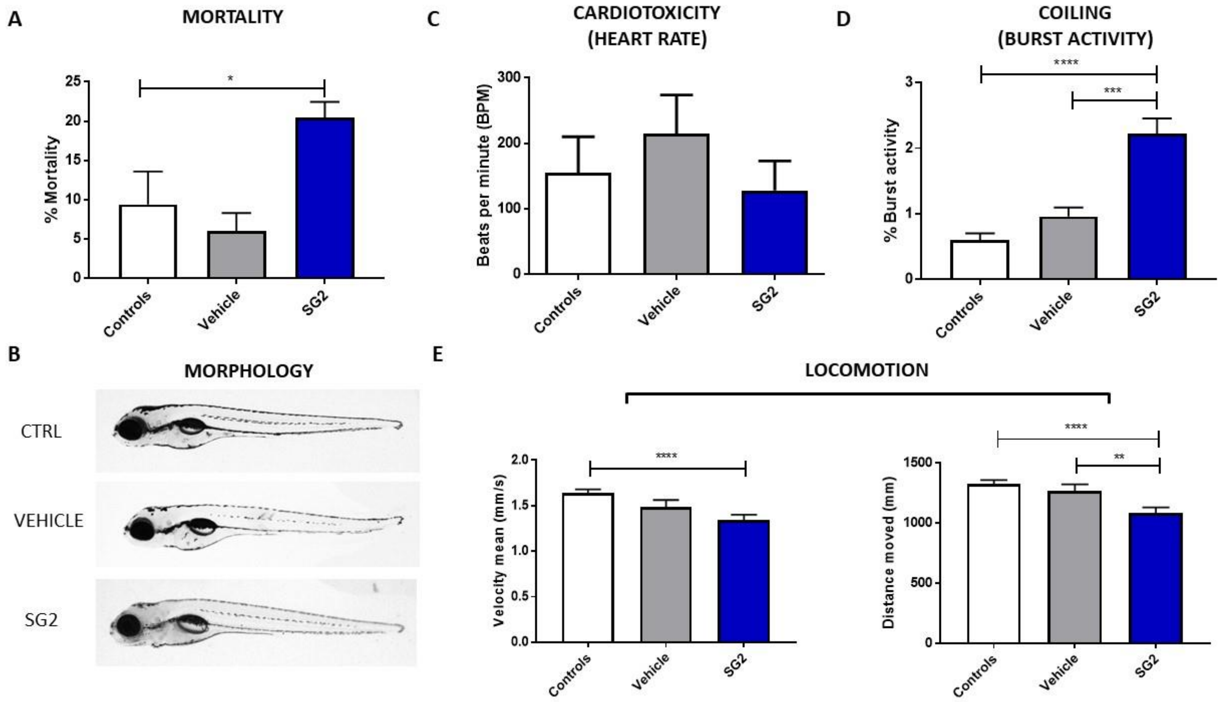

Figure S5. SG2 10µM effects in zebrafish. SG2 was administered at 10 µM to the zebrafish at 4 hpf. This dose revealed a slightly toxic effect on zebrafish neurodevelopment. Mortality rate was calculated until 48 hpf (A) (controls N=220, vehicle N=143, SG2 N=141) and no morphological anomaly was observed at 5 dpf (B). Cardiotoxicity was evaluated measuring heart rate at 3 dpf (C). Neurological development was evaluated at 30 hpf with burst activity analysis (D) and at 5 dpf with locomotor measurements considering velocity and distance covered (E) (controls N=345, vehicle N=131, SG2 N=249). All experiments were performed at least in triplicate. Error Bars represent Standard Error on the Mean (SEM). For statistical tests, non-parametric one-tailed Mann-Whitney rank sum test was used. *p≤0.05, **p≤0.01, ***p≤0.001, or ****p≤0.0001. Only significant data are reported in all the graphs.

Table S1. Primer sequences used in real time PCR experiments.

Gene	Accession Number		Primers
**MTOR**	NM_004958	*forward*	**5’ TGCCTTCACAGATACCCAGTA 3’**
		reverse	**5’ AGACCTCACAGCCACAGA 3’**
**SIGMAR1**	NM_005866	*forward*	**5’ TATACTCTTCGCTCCTAT 3’**
		reverse	**5’ TATGTCCCTGTCTGTAAA 3’**
**SIRT1**	NM_012238	*forward*	**5’GTAGGCGGCTTGATGGTAAT 3’**
		reverse	**5’ GGGTTCTTCTAAACTTGGACTCT 3’**
**SIRT5**	NM_012241	*forward*	**5’ AATAACTAAAGCCCGCCTCAA 3’**
		reverse	**5’ CAAATCTGGTTTCGTGTGGAC 3’**
**SIRT6**	NM_016539	*forward*	**5’ CTCCTCCGCTTCCTGGTC 3’**
		reverse	**5’ TTACACTTGGCACATTCTTCC 3’**
**LC3B**	NM_022818	*forward*	**5’ AGTCTTCTCTTCAGGTTCAC 3’**
		reverse	**5’ CTCACACAGCCCGTTTAC 3’**
**GAPDH**	NM_002046	*forward*	**5’ CCCTTCATTGACCTCAACTACATG 3’**
		reverse	**5’ TGGGATTTCCATTGATGACAAGC 3’**
